# Transplantation of vascularized cardiac microtissue from human induced pluripotent stem cells improves impaired electrical conduction in a porcine myocardial injury model

**DOI:** 10.1016/j.xjon.2025.03.006

**Published:** 2025-03-17

**Authors:** Yuki Kuroda, Jun Iida, Kozue Murata, Yuki Hori, Jumpei Kobiki, Kenji Minatoya, Hidetoshi Masumoto

**Affiliations:** a Department of Cardiovascular Surgery, Graduate School of Medicine, Kyoto University, Kyoto, Japan; b Clinical Translational Research Program, RIKEN Center for Biosystems Dynamics Research, Kobe, Japan

**Keywords:** induced pluripotent stem cell, cardiac regenerative therapy, heart failure, cell transplantation

## Abstract

**Objective:**

To demonstrate that the transplantation of human induced pluripotent stem cell (hiPSC)-derived vascularized cardiac microtissue (VCM) can improve conduction disturbances after myocardial injury (MI).

**Methods:**

We prepared cell sheet-shaped VCM with hiPSC-derived cardiomyocytes and vascular cells using dynamic rocking culture. We induced MI via epicardial cryoablation in immunosuppressed crown minipigs (VCM and sham groups; n = 3) and transplanted the VCMs immediately after MI induction. The pigs underwent epicardial electroanatomical mapping immediately before and 1 week after MI induction.

**Results:**

One week after MI induction, mean electrical potentials at the MI site decreased in both groups during sinus rhythm (from 11.05 to 1.74 mV in the VCM group and from 8.72 to 2.70 mV in the sham group, *P* = .048). The mean conduction velocity between the remote and MI sites was numerically higher in the VCM group compared with the Sham group (2.84 m/s vs 1.74 m/s). One of the 3 animals in the VCM group demonstrated 2 independent origins of excitation corresponding to the pacing sites when simultaneous pacing of the remote and MI sites was performed 1 week after MI induction. Histologic examination confirmed that the VCM had engrafted on the surface of the MI region. Furthermore, we confirmed that the myocardial tissue in the MI region remained more intact one week after injury in the VCM transplantation group compared to the sham group, suggesting that this contributed to the reduction of conduction disturbances.

**Conclusions:**

The transplantation of VCM demonstrated a potential for improving conduction disturbances in MI.

## Graphical Abstract

**Figure:**
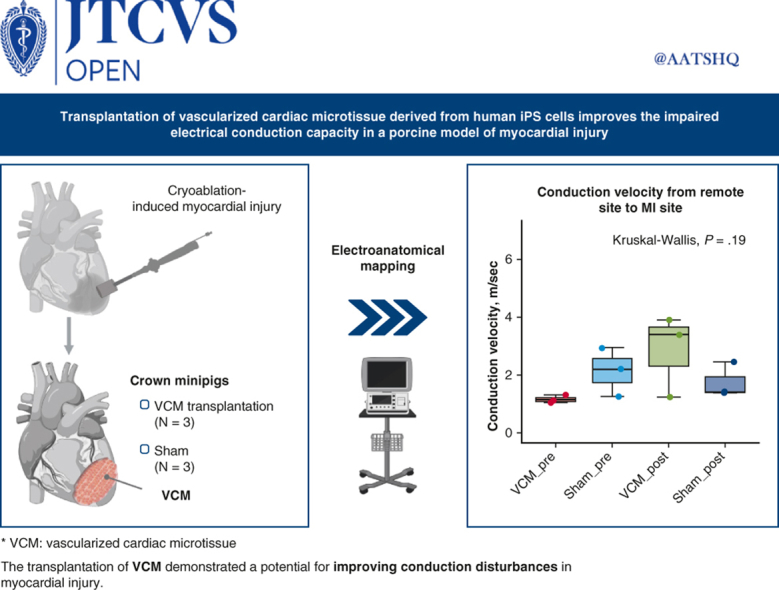

**Figure:**
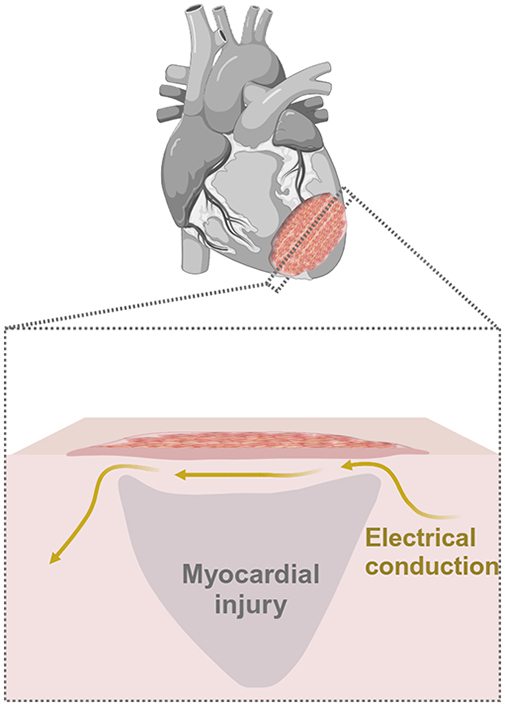
VCM transplantation improved conduction disturbances in a porcine myocardial injury model.

**Central Message**

The transplantation of VCM improved conduction disturbances in the porcine MI model. Epicardial EAM may serve as an indicator for restoration of the myocardial layer in clinical settings.

**Perspective**

This study demonstrated that the transplantation of hiPSC-derived VCM can improve electrophysiological disturbances caused by myocardial injury in a pig model. This indicates the electrophysiological efficacy and safety of transplanting 3-dimensional cardiac tissue constructs derived from hiPSCs, which may serve as evidence of electrophysiological advantage in stem cell-based cardiac cell therapy.

The management of severe heart failure has evolved greatly in recent years, including medications, mechanical circulatory support, and cardiac transplantation.^1^ Although mechanical circulatory support and cardiac transplantation are effective treatments for patients who develop persistent severe symptoms despite maximum medical therapy, there are still issues to be solved, such as device-related complications and shortage of donor hearts.^2^^,^^3^

Pluripotent stem cell-based (eg, embryonic stem cells, induced pluripotent stem cells [iPSCs]) cardiac regenerative therapy is expected to be a potential treatment modality for severe heart failure.^4^^,^^5^ Several clinical trials of cardiac regenerative therapy using pluripotent stem cells have been conducted.^6^^,^^7^ However, in order to achieve effective regenerative medicine, optimal grafts should have a morphology and structure that is close to the living heart, maintaining viability and achieving electrical and mechanical synchronization with the host heart.^8^

To overcome the unsatisfactory results of most clinical research on stem cell injections to an injured heart, we developed bioengineered cardiac tissue grafts using human induced pluripotent stem cell (hiPSC)-derived cardiomyocytes and vascular cells.^9^^,^^10^ Temperature-responsive culture dishes allowed us to collect cell sheets by simply decreasing the temperature and without enzymatic digestion.^11^ Furthermore, dynamic rocking culture with cyclic see-sawing stimuli using digital rockers has been reported to promote maturation of hiPSC-derived engineered heart tissues.^12^^,^^13^ This technique allowed us to produce thick, more mature cardiac tissue sheets with vascular structure, called vascularized cardiac microtissue (VCM).^14^^,^^15^

There is a scarcity of data regarding changes in electrical excitation propagation and electrical potentials, as well as responsiveness to pacing stimuli in the diseased host heart when hiPSC-derived heart tissue is transplanted. Therefore, in this study, we aimed to demonstrate that transplantation of hiPSC-derived VCM can improve conduction disturbances in the impaired heart using a porcine myocardial injury (MI) model. In addition, we investigated whether simultaneous pacing of healthy and posttransplantation lesion areas could further improve this conduction disturbance.

## Methods

### Experimental Study Design

All animal experiment protocols were approved by the Animal Experimentation Committee of Kyoto University (Med Kyo 23158; March 24, 2023) and performed according to the Guidelines for Animal Experiments of Kyoto University, which conform to Japanese law and the US National Research Council's Guide for the Care and Use of Laboratory Animals.

**Figure 1:**
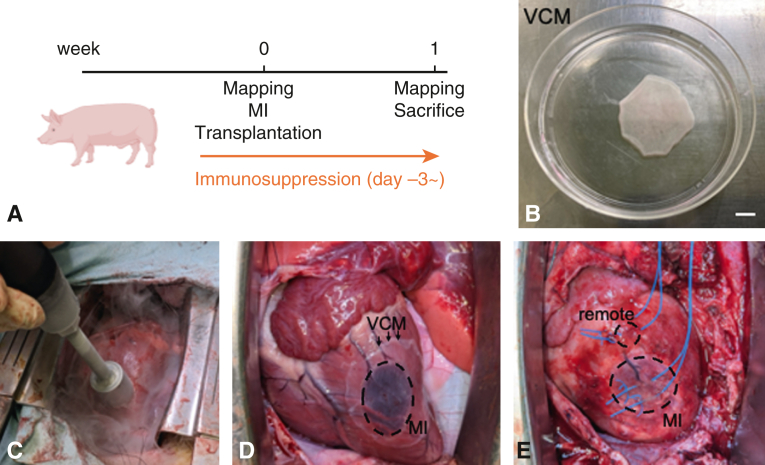
Experimental methods. A, Time course of the animal experiments. B, VCM sheet on the 10-cm UpCell dish (Scale bar: 1 cm). C, MI induction via cryoablation. D, VCM covering the MI area. E, Epicardial pacing. *VCM*, Vascularized cardiac microtissue; *MI*, myocardial injury.

Six female crown minipigs (Kagoshima Miniature Swine Research Center) weighing 15 to 25 kg were randomly divided into 2 groups: VCM transplantation (VCM group, n = 3) and control (sham group, n = 3). Female minipigs were selected for this experiment because of their ease of handling during the procedures. All animals underwent MI induction via cryoablation. VCMs were transplanted immediately after MI induction in the VCM group. Electroanatomical mapping (EAM) was performed just before and 1 week after MI induction. After the second EAM, the animals were killed humanely by intravenous bolus injection of potassium chloride (1-2 mmol/kg), and those hearts were harvested for histologic analysis (Figure 1, *A*).

### Preparation of VCMs

In the present study, we used human iPSCs lines 201B6 established at the Center for iPS Cell Research and Application, Kyoto University.^16^ We induced cardiomyocytes, endothelial cells, and vascular mural cells from hiPSCs as previously reported.^10^^,^^17^^,^^18^ The induced multiple cardiovascular cells were dissociated by incubation with Accutase (Innovative Cell Technologies), and the cell population was measured by flow cytometry of the mixture of cells. The cell mixture was plated onto a fetal bovine serum−coated 10-cm dish UpCell plate (CellSeed) at 20,000,000 cells/well with 17 mL of attachment medium (alpha minimum essential medium from Thermo Fisher Scientific supplemented with 10% fetal bovine serum and 5 × 10^−5^ M of 2-mercaptoethanol) containing 25 ng/mL vascular endothelial growth factor and 10 μM Y-27632. After 2 days in culture, 25 ng/mL vascular endothelial growth factor was added to the culture medium. After 2 more days in culture, the cells were moved to room temperature. Within 15 minutes, the cells detached and floated in the media as monolayer cardiac tissue sheets. The collected cardiac tissue sheets were allowed to reattach to Matrigel-coated 10-cm dishes and incubated with attachment medium containing 10 μM Y-27632 for 24 hours. Then, we used Compact Digital Rocker (Thermo Fisher) at 60 rpm and 13° for 14 days to thicken the cardiac microtissue and prepare VCM composed of cardiomyocytes and vascular cells (Figure 1, *B*).^15^

### Porcine Models and Protocols for MI Induction and VCM Transplantation

All animals received immunosuppression with daily administration of tacrolimus (0.75 mg/kg), mycophenolate mofetil (500 mg), and prednisolone (20 mg) from 3 days before transplantation until they were killed humanely. For all surgical procedures, the pigs were premedicated with an intramuscular injection of ketamine hydrochloride (20 mg/kg), xylazine hydrochloride (2 mg/kg), and atropine sulfate (0.5 mg) and intubated endotracheally. General anesthesia was maintained with 5 L/min of O_2_ and isoflurane (1%-2%). Cefazolin sodium (500 mg) was administered intravenously before skin incision. Amiodarone (75 mg) was administered intravenously before pericardium incision to prevent ventricular arrhythmia. The animals were positioned in the right lateral decubitus position, and percutaneous oxygen, electrocardiogram, and intermittent blood pressure were monitored.

The pericardial space was exposed through left thoracotomy. MI was induced by cryoablation with a stainless rod 2 cm in diameter frozen in liquid nitrogen (Figure 1, *C*, and Video 1). The cryo-rod was applied to the anterior free wall of the left ventricle at 1 cm lateral to the left anterior descending artery (to avoid the injury of the left anterior descending artery) for 2 minutes, moving concurrently with heart beats. After MI induction, we transplanted VCMs treated with Hoechst 33342 (Thermo Fisher), covering the cryoablation-induced MI area (Figure 1, *D*, and Video 2), and secured the sheet circumferentially with 6-0 polypropylene sutures. The pericardium was closed with a 0.1-mm expanded polytetrafluoroethylene membrane (W. L. Gore & Associates). In the sham group, MI was induced by cryoablation, and the area was then covered with a 0.1-mm expanded polytetrafluoroethylene membrane. In all 6 pigs, except for premature beats observed during cardiac manipulation, no premature beats, atrial flutter, or atrial fibrillation were observed during the surgical procedures. During the surgery, no obvious signs of diastolic dysfunction indicating pericarditis were observed macroscopically in all animals.

### Electroanatomical Mapping

We used CARTO3 (Biosense Webster) and a 7-F mapping catheter (DECANAV; Biosense Webster) with 10 electrodes. EAM of the left ventricle was performed from the epicardial side after pericardial incision under sinus rhythm and epicardial pacing. Epicardial pacing was performed in the following patterns using an electric voltage generator (USE-210; Unique Medical): remote site pacing, and simultaneous remote and MI site pacing to evaluate how electrical stimulation to the VCM influences conduction in the host heart (Figure 1, *E*). Differences in myocardial electrical potentials and conduction velocities were assessed between VCM and sham groups.

### Histologic Analysis

Heart tissues from the left ventricle were fixed in 4% paraformaldehyde and embedded in paraffin. Sections were prepared along the short axis and stained with hematoxylin-eosin and immunostaining for cardiac troponin T (cTnT). We confirmed engrafted cells with Hoechst 33342 staining, which was applied to VCM before transplantation. For cTnT staining, paraffin sections were stained first with a mouse monoclonal antibody for cTnT (Thermo Fisher) (1:100). They were incubated with a biotinylated secondary antibody (1:300) and avidin-biotin-peroxidase complex (ABC-Elite, Vector Laboratories, 1:100). Coloring reaction was carried out with DAB and nuclei were counterstained with hematoxylin. Images were photographed using an all-in-one digital microscope (BZ-X810; Keyence). The ratio of preserved cardiomyocytes in the MI area was calculated by dividing the area of remaining cardiomyocytes by the total area of the MI area.

### Statistical Analysis

Electrical potentials and conduction velocities were presented as mean ± standard deviation and were compared between the groups using the Kruskal-Wallis test. Percentages of the area of preserved cardiomyocytes out of MI area were compared between the groups using the Wilcoxon rank-sum test. All statistical analyses were conducted using R statistical software, version 4.4.1 (R Foundation for Statistical Computing). All reported *P* values were 2-tailed.

## Results

### Electrical Potential

**Figure 2:**
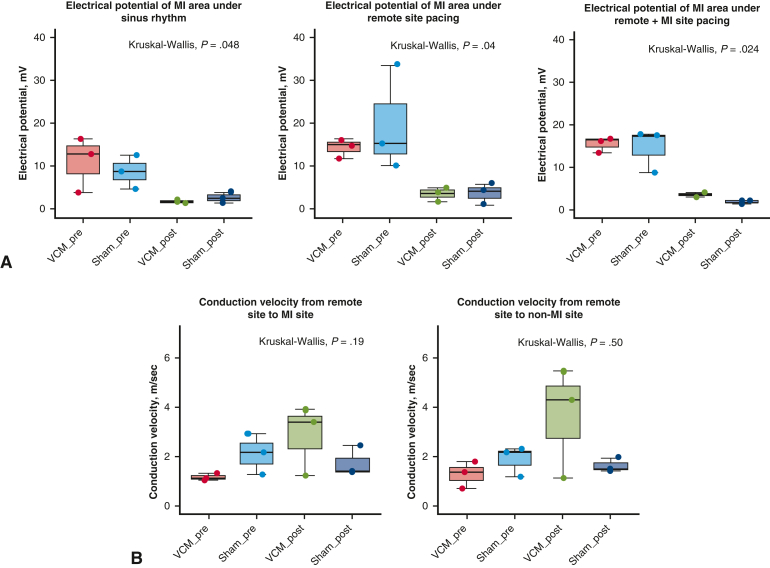
Impact of VCM transplantation on electrical potential and conduction velocity. A, Electrical potentials of MI site under sinus rhythm, remote site pacing, and simultaneous remote and MI site pacing before (VCM_pre, Sham_pre) and 1 week after MI induction (VCM_post, Sham_post). B, Conduction velocities under remote pacing before (VCM_pre, Sham_pre) and 1 week after MI induction (VCM_post, Sham_post) (remote site to MI site and remote site to non-MI site). The *upper and lower borders of the box* indicate the upper and lower quartiles; the *middle horizontal line* represents the median; and the *upper and lower whiskers* show the maximum and minimum values in all box-and-whiskers graphs. *VCM*, Vascularized cardiac microtissue; *MI*, myocardial injury.

First, in order to evaluate the impact of VCM transplantation on electrical potentials in injured myocardium, we measured mean electrical potentials in the MI area under sinus rhythm, remote site pacing, and simultaneous remote and MI site pacing. One week after MI induction, mean electrical potentials at the MI area decreased in both groups during sinus rhythm (from 11.05 ± 6.50 mV to 1.74 ± 0.36 mV in the VCM group, and from 8.72 ± 3.91 mV to 2.70 ± 1.25 mV in the sham group, *P* = .048) (Figure 2, *A*). Mean electrical potentials at the MI area decreased in both groups during remote site pacing (from 14.26 ± 2.35 mV to 3.44 ± 1.63 mV in the VCM group, and from 19.64 ± 12.29 mV to 3.64 ± 2.43 mV in the sham group, *P* = .04). Mean electrical potentials at the MI area decreased in both groups during simultaneous remote and MI site pacing (from 15.61 ± 1.81 mV to 3.67 ± 0.62 mV in the VCM group, and 14.66 ± 5.17 mV to 1.96 ± 0.45 mV in the sham group, *P* = .024). The electrical potential 1 week after MI was almost the same between the sham group and the VCM group across all experimental conditions. From these results, we confirmed that the electrical potential decreased as the result of the MI induction, whereas the VCM transplantation did not restore the electrical potential in the injured myocardium.

### Conduction Velocity

**Figure 3:**
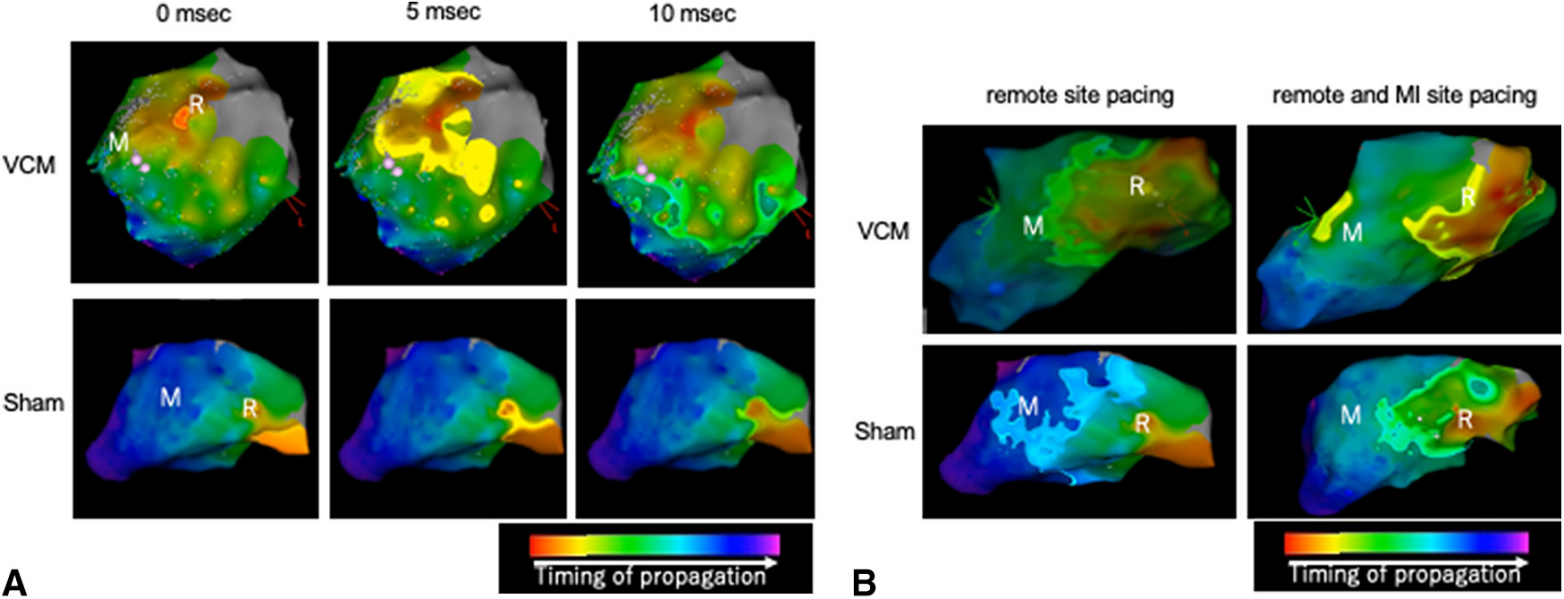
Excitation propagation visualized by the electroanatomical mapping. A, Representative images of electroanatomical mapping (*EAM*) under remote pacing 1 week after myocardial injury (*MI*) induction (R: remote site, M: MI site). B, Representative images of EAM under simultaneous remote and MI site pacing 1 week after MI induction (R: remote site, M: MI site). *VCM*, Vascularized cardiac microtissue.

Next, we measured mean conduction velocities from remote to remote and MI site in order to evaluate the capacity of VCM transplantation to improve excitation propagation. The mean conduction velocity between the remote and MI sites was numerically greater in the VCM group compared with the sham group (2.84 ± 1.42 m/s v 1.74 ± 0.61 m/s) (Figure 2, *B* and Figure 3, *A*, and Video 3). In addition, it increased 1 week after MI induction in the VCM group (from 1.16 ± 0.14 m/s to 2.84 ± 1.42 m/s), whereas it did not in the sham group (from 2.13 ± 0.84 m/s to 1.74 ± 0.61 m/s). Similarly, the mean conduction velocity between the remote and non-MI (a point of the same distance in the noninjured area as MI site from the remote site) sites was numerically greater in the VCM group (3.63 ± 2.24 m/s vs 1.62 ± 0.29 m/s), and it increased 1 week after MI induction in the VCM group (from 1.27 ± 0.55 m/s to 3.63 ± 2.24 m/s), whereas it did not in the sham group (from 1.88 ± 0.63 m/s to 1.62 ± 0.29 m/s). These results suggest that VCM transplantation has the potential to improve conduction disturbances caused by MI.

### Simultaneous Remote and MI Site Pacing

Next, we performed simultaneous pacing of the remote and infarcted (VCM transplanted) sites to confirm whether electrophysiological synchronization between the grafts and the host myocardium could be achieved. One of the 3 animals in the VCM group demonstrated 2 independent origins of excitation corresponding to the pacing sites when simultaneous pacing of the remote and MI sites was performed 1 week after VCM transplantation, while the origin of conduction in the MI site was not observed in other animals, including those in the sham group (Figure 3, *B*, and Video 4). Although not conclusive, this result suggests that simultaneous pacing may help to achieve synchronization between the grafts and host myocardium when VCM is transplanted to the injured myocardium.

### Histologic Analysis

**Figure 4:**
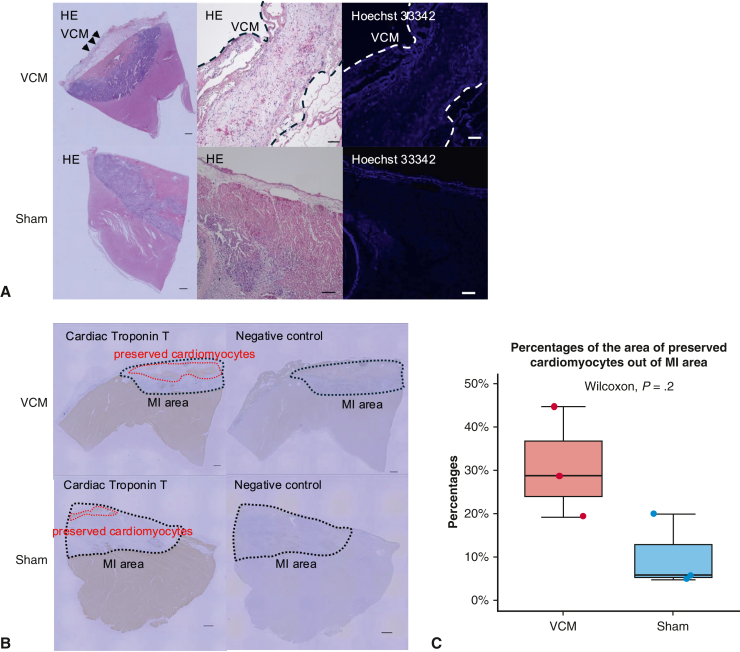
Engraftment of VCMs and the preservation of host myocardium after VCM transplantation. A, Representative hematoxylin eosin (*HE*) staining (*dark purple*: cell nuclei) and Hoechst 33342 staining (scale bars: 1 mm for the leftmost panels and 100 μm for other panels.) B, Representative immunostaining for cardiac troponin T (*brown*: cardiomyocytes, *red dotted lines*: cardiomyocytes in the MI area) and negative control of immunostaining (scale bars: 1 mm). C, Percentages of the area of preserved cardiomyocytes out of MI area. The *upper and lower borders of the box* indicate the upper and lower quartiles; the *middle horizontal line* represents the median; and the *upper and lower whiskers* show the maximum and minimum values in all box-and-whiskers graphs. *VCM*, Vascularized cardiac microtissue; *MI*, myocardial injury.

Finally, we conducted histologic analysis to confirm how the electrophysiological abnormalities were improved by the transplantation of VCM. Hematoxylin-eosin and Hoechst 33342 staining revealed that the VCM had engrafted on the surface of the MI region 1 week after transplantation (Figure 4, *A*). cTnT staining revealed that the myocardial tissue in the MI region remained more intact 1 week after MI in the VCM transplantation group compared with the sham group, and the percentage of the area of preserved cardiomyocytes out of MI area was numerically greater in the VCM group than in the sham group (31 ± 13% vs 10 ± 9%, *P* = .2), suggesting that this contributed to the reduction of conduction disturbances (Figure 4, *B*, and *C*).

## Discussion

**Figure 5:**
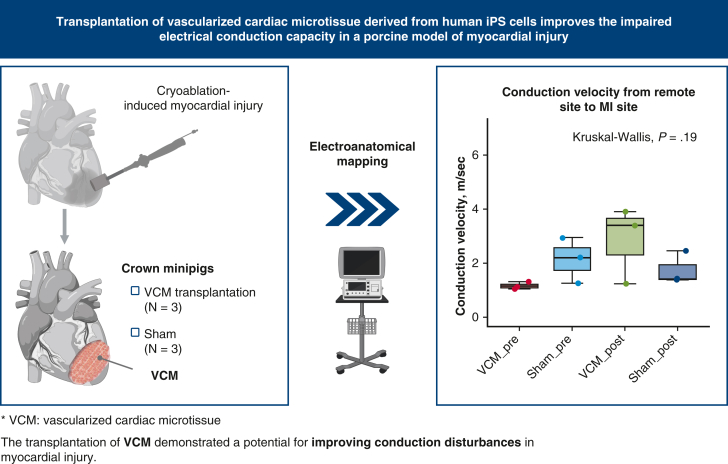
Visualization of methods and key findings of this experiment. *iPSC*, Induced pluripotent stem cell; *VCM*, vascularized cardiac microtissue; *MI*, myocardial injury.

The VCM group demonstrated faster conduction velocity compared with the sham group (Figure 5). This result suggests that the transplantation of VCM onto the surface of the MI region functionally improved the electrical conduction disturbance. Histologically, there was a greater preservation of the myocardium in the VCM-covered MI region 1 week after transplantation. This indicates that VCM transplantation may have suppressed the progression of cardiomyocyte loss, which occurs as part of the natural course after cryoinjury. Although the VCM used in this study was much thinner than porcine myocardium, the results indicated that even such a thin tissue has the potential to improve electrical conduction disturbances, which directly affects global cardiac function and the attenuation of posttransplant arrhythmia, when it is transplanted to cover the injured area. This can be viewed as a positive outcome for future stem cell−based cardiac treatments.

Further studies are needed to investigate the mechanism behind the preservation of host myocardium mediated by VCM transplantation. A previous study has reported that transplantation of iPS cell-derived cardiomyocytes improved cardiac function in mouse MI models through paracrine effects, including antiapoptotic effects, proangiogenesis, and progenitor cell migration mediated by various cytokines.^19^ The VCM used in our experiment is a myocardial tissue sheet containing vascular structures,^15^ which we believe promotes angiogenesis in the host heart more effectively. In contrast, we consider the possibility that cardiomyocytes play a role in electrical conduction and that better preservation of cardiomyocytes in the infarcted area in the VCM group may have contributed to improved conduction. Identifying and evaluating specific cells involved in the formation of the conduction system remains a challenge for future research. Our experimental model was designed to understand the electrophysiological aspects of VCM therapy and did not have a sufficient observation period to evaluate cardiac functional recovery or left ventricular remodeling. However, for the broader adoption of this therapy, it is important to not only investigate its electrophysiological effects but also develop a comprehensive understanding on the basis of cardiac physiology.

Although engraftment of VCM on the surface of the injured area was also confirmed, it was not fully elucidated in this study whether the VCM itself contributed to physically alleviating the conduction disturbance. Considering that this study did not clearly demonstrate the effect of VCM transplantation on the increase of electrical potential in the injured area (Figure 2, *A*), it is possible that further development and transplantation of grafts with greater thickness and structures more closely resembling those of living hearts may be required for the transplanted VCM to physically alleviate the conduction disturbances and promote myocardial regeneration. This is considered a subject for future research and development.

Another important aspect of enhancing the electrophysiological benefits of cardiac regenerative medicine using hiPSCs is improving the maturity of hiPSC-derived cardiomyocytes. HiPSC-derived cardiomyocytes are generally considered immature with lower electrical conduction capacity than mature cardiomyocytes.^20^^,^^21^ There are several reports regarding the electrical coupling between the stem cell−derived graft and the host heart. Intracardiac injection of human embryonic stem cell−derived cardiomyocytes in a cryoinjured guinea pig model have been reported to show 1:1 electrical coupling between hESC-derived cardiomyocytes and host myocardium.^8^ HiPSC-derived cardiomyocytes in the spheroids have also been reported to electrically couple with the host myocardium at rates up to 240 beats/min.^22^ In contrast, there are few reports regarding the electrical coupling between 3-dimensional tissue constructs derived from hiPSCs and the host myocardium. Should the transplanted cardiac tissue structures and the cardiomyocytes that compose them become more mature, further improvement in conduction disturbances, including electrical potentials in the myocardial infarction area, can be expected. In the research and development process aimed at achieving this improvement, the EAM used in this study could serve as an indicator for the regeneration of the myocardium and the improvement of electrophysiological functions in preclinical and clinical settings. As a separate result, we observed that one of the pigs in the VCM group showed 2 origins of excitation under simultaneous remote and MI site pacing (Figure 3, *B*). Although this result did not directly demonstrate the electrical coupling between the graft and host myocardium, simultaneous pacing of the transplanted and remote sites may offer a potential solution to enhance the electrical coupling.

This study has several limitations. First, because of the xenotransplantation between pigs and human cell-derived cardiac tissue sheets and possible immune rejection even administrating immunosuppressants, we may underestimate the effectiveness of VCM transplantation. Furthermore, the timing of VCM transplantation after MI, as well as the administration of immunosuppressants and antiarrhythmic drugs, does not fully reflect actual clinical conditions. In this study, we believe that we have at least demonstrated proof of concept that VCM can suppress electrical conduction disturbances. However, these results are solely on the basis of an experimental setting of xenogeneic transplantation. The effectiveness of VCMs for treating electrical conduction disturbances in humans should be further validated in future clinical studies. Second, cryoablation-induced MI and clinical myocardial infarction might have fundamentally different mechanisms for causing conduction disturbances. However, a previous report have shown that models using ameroid constrictors and cryoablation produce similar patterns of fibrosis.^23^ When using ameroid constrictors, it is more difficult to control the infarct size. Since our study aimed to ensure reliable coverage of the MI area, we used cryoablation, which allows creation of MI within a defined region. In addition, cryoablation was suitable for epicardial electroanatomical mapping, because it can create myocardial injury from the epicardial side. Third, this study is underpowered for conducting nonparametric tests with three-by-three comparisons. However, we designed this as an exploratory study, aiming to interpret individual data obtained as measurements and phenomena rather than to demonstrate statistical significance.

## Conclusions

The transplantation of VCM derived from hiPSCs demonstrated a potential for improving conduction disturbances in a porcine MI model. This research may have provided electrophysiological evidence supporting the efficacy of regenerative medicine using hiPSCs for myocardial injury.

### Declaration of Generative AI and AI-Assisted Technologies in the Writing Process

During the preparation of this work the author(s) used ChatGPT in order to improve language and readability, with caution. After using this tool/service, the author(s) reviewed and edited the content as needed and take(s) full responsibility for the content of the publication.

## Conflict of Interest Statement

K. Murata and H.M. are inventors of a related patent. All other authors reported no conflicts of interest.

The *Journal* policy requires editors and reviewers to disclose conflicts of interest and to decline handling or reviewing manuscripts for which they may have a conflict of interest. The editors and reviewers of this article have no conflicts of interest.

## Supplementary Data

Video 1 Cryoablation with a stainless rod frozen in liquid nitrogen. Video available at: https://www.jtcvs.org/article/S2666-2736(25)00078-6/fulltext.

Video 2 Transplantation of vascularized cardiac microtissue. Video available at: https://www.jtcvs.org/article/S2666-2736(25)00078-6/fulltext.

Video 3 Representative movies of electroanatomical mapping under remote pacing 1 week after myocardial injury induction. Video available at: https://www.jtcvs.org/article/S2666-2736(25)00078-6/fulltext.

Video 4 Representative movies of electroanatomical mapping under simultaneous remote and myocardial injury (MI) site pacing 1 week after MI induction. Video available at: https://www.jtcvs.org/article/S2666-2736(25)00078-6/fulltext.
