# Supplementary figures and images for: Transplantation of vascularized cardiac microtissue from human induced pluripotent stem cells improves impaired electrical conduction in a porcine myocardial injury model
Source: JTCVS Open. 2025 Mar 17;25:154–62. doi: 10.1016/j.xjon.2025.03.006 (PMC12230479; doi:10.1016/j.xjon.2025.03.006)

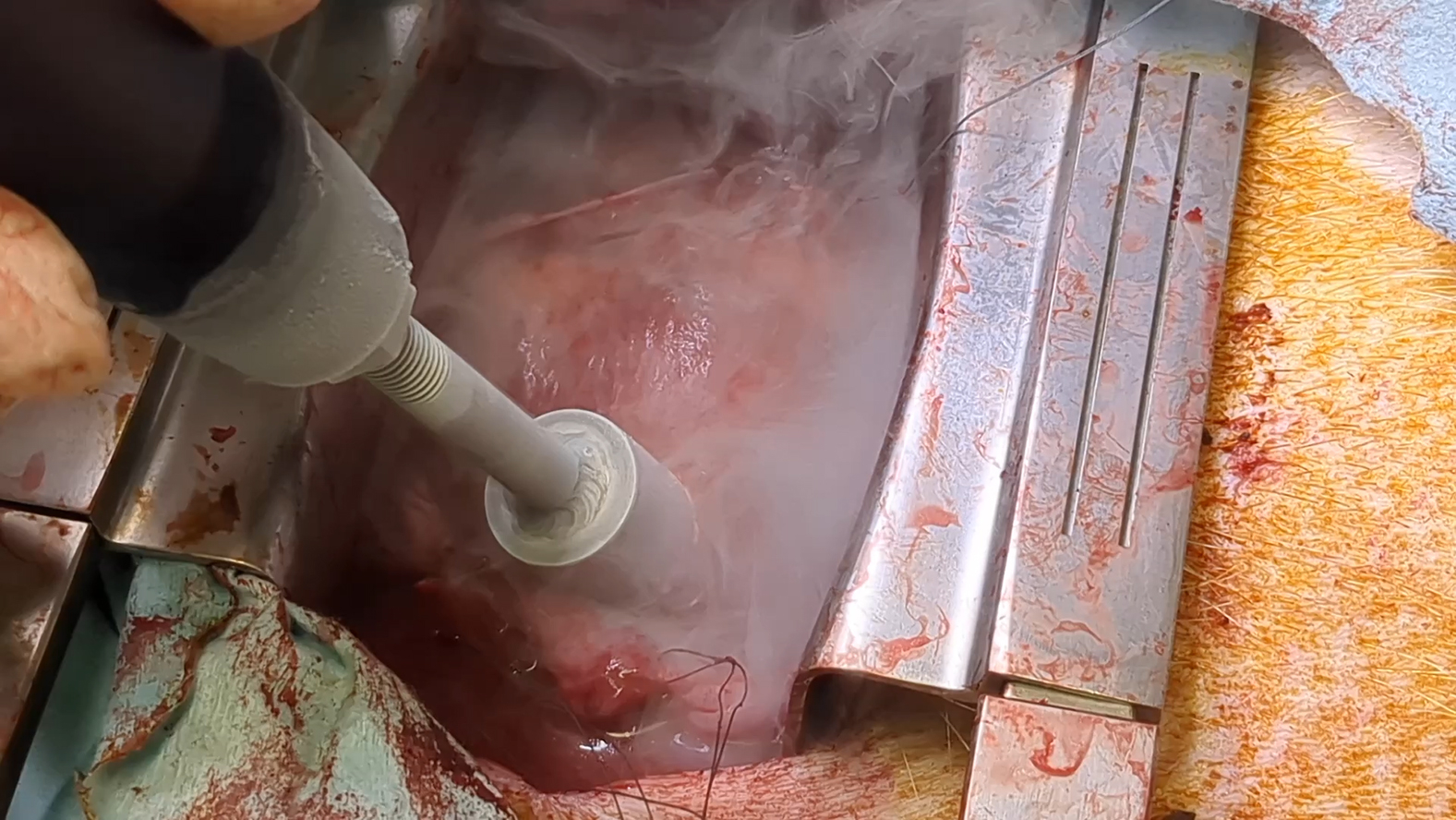

Supplement: Video 1 — Cryoablation with a stainless rod frozen in liquid nitrogen. Video available at: https://www.jtcvs.org/article/S2666-2736(25)00078-6/fulltext. [file fx2.jpg]

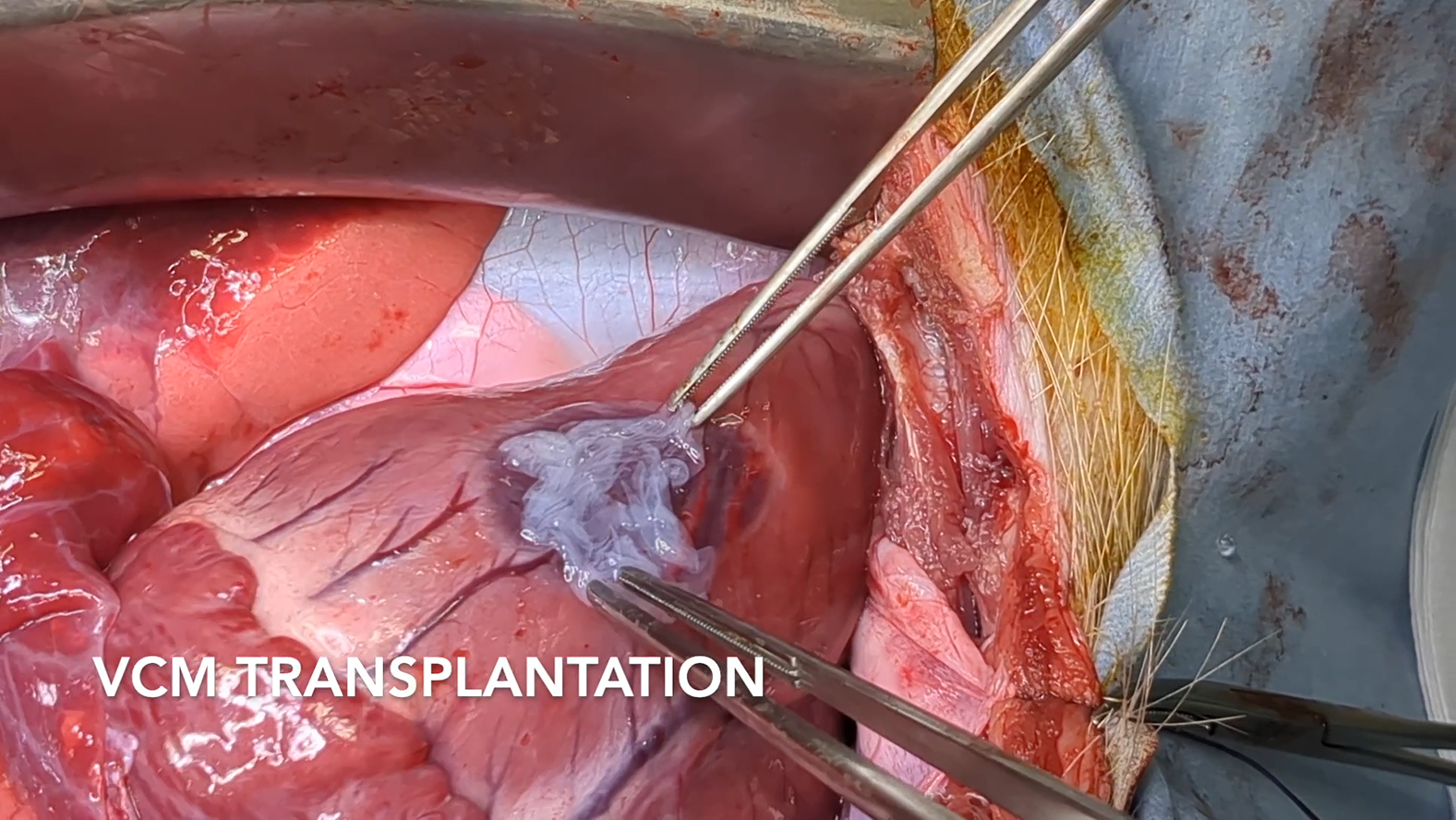

Supplement: Video 2 — Transplantation of vascularized cardiac microtissue. Video available at: https://www.jtcvs.org/article/S2666-2736(25)00078-6/fulltext. [file fx3.jpg]

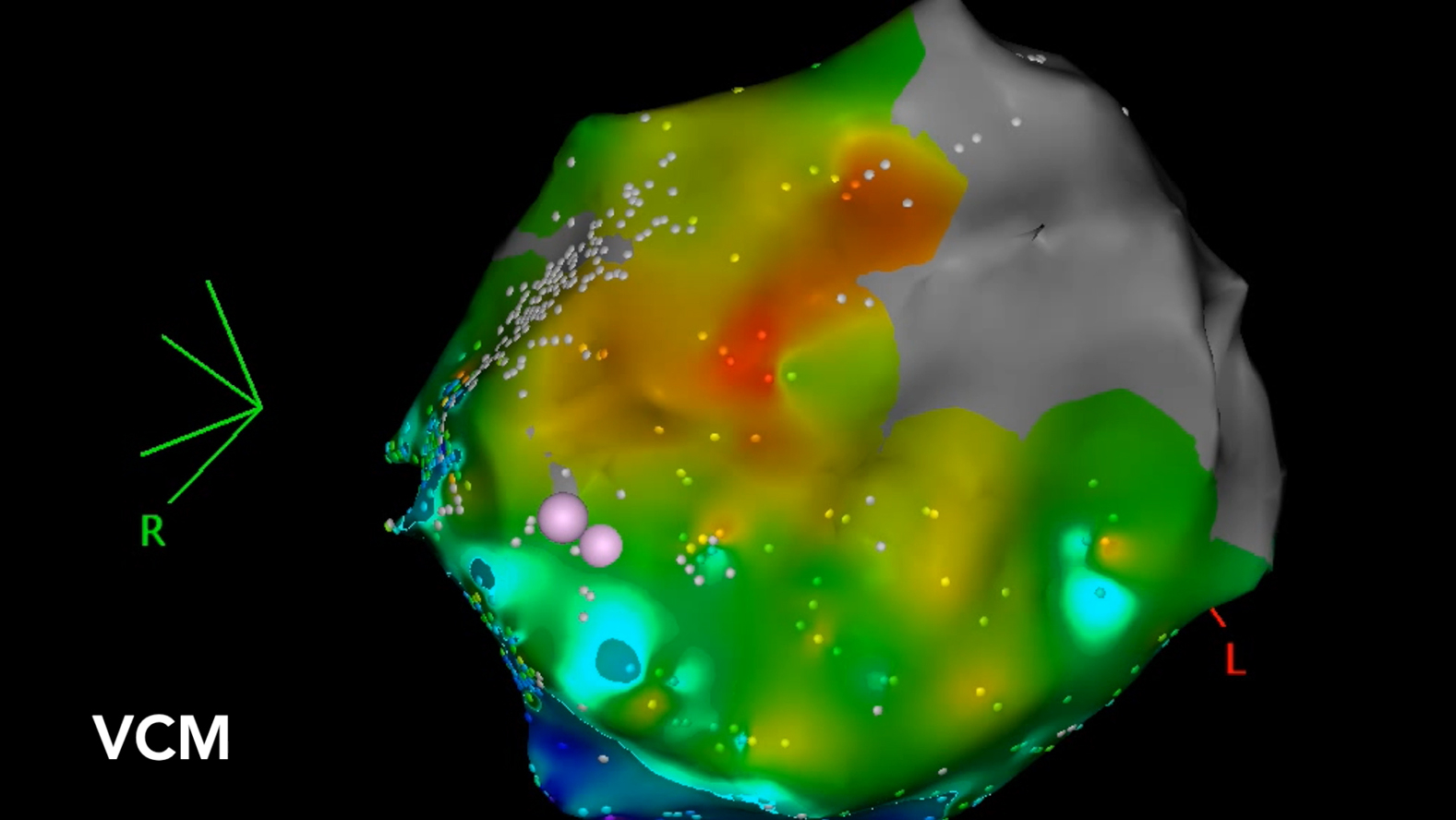

Supplement: Video 3 — Representative movies of electroanatomical mapping under remote pacing 1 week after myocardial injury induction. Video available at: https://www.jtcvs.org/article/S2666-2736(25)00078-6/fulltext. [file fx4.jpg]

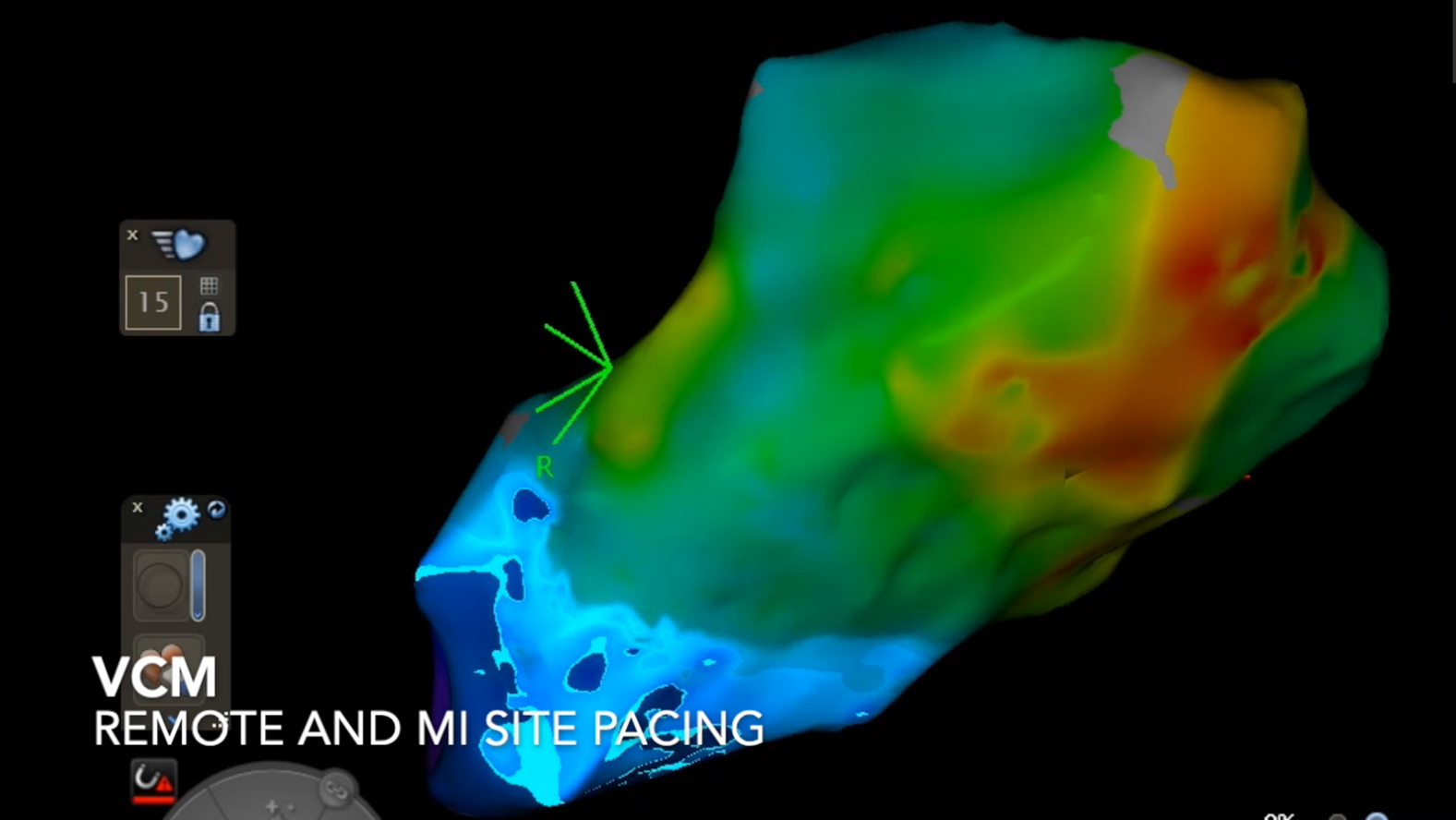

Supplement: Video 4 — Representative movies of electroanatomical mapping under simultaneous remote and myocardial injury (MI) site pacing 1 week after MI induction. Video available at: https://www.jtcvs.org/article/S2666-2736(25)00078-6/fulltext. [file fx5.jpg]
